# Post remote ischaemic conditioning and early pre remote ischaemic conditioning reduces the extent and severity of bowel injury in an animal model of necrotising enterocolitis

**DOI:** 10.1007/s00383-026-06717-8

**Published:** 2026-11-13

**Authors:** Ian Howard Jones, Denise Tao, Bhumita Vagdama, Laurie Lau, Jane Collins, Nigel John Hall

**Affiliations:** 1 Department of Paediatric Surgery and Urology, Birmingham Children’s Hospital, Birmingham, UK; 2 University Surgery Unit, Faculty of Medicine, University of Southampton, Tremona Road, Southampton, SO16 6YD UK; 3 Department of Histopathology, University Hospitals Southampton NHS Foundation Trust, Southampton, UK

**Keywords:** NEC, Necrotising enterocolitis, Intestinal ischaemia, RIC, Remote ischaemic conditioning

## Abstract

**Purpose:**

Remote ischaemic conditioning (RIC) has a protective effect in an experimental model of severe necrotising enterocolitis. We investigated the impact of delivering RIC at different time-points.

**Methods:**

We used an established intestinal ischaemia-reperfusion injury (IRI) model in rat pups. RIC was delivered by means of a lower limb ligature. Animals were randomly allocated to intestinal IRI only, RIC before IRI (preRIC), RIC after IRI (postRIC), RIC 48 h before IRI (earlyRIC), RIC 48 h and immediately before IRI (early + preRIC), or control. Macroscopic and microscopic intestinal injury are reported. Serum cytokine levels were assayed. Data are median (inter-quartile range).

**Results:**

Animals receiving RIC had reduced severe macroscopic intestinal injury: pre-RIC 4% (0–21%, *p* = 0.0019), postRIC 4% (0–44%, *p* = 0.0021), earlyRIC 3% (0–42%, *p* = 0.0054) and early+preRIC 0% (0–39%, *p* = 0.0018) vs. IRI 78% (30–90). The preRIC and postRIC groups compared had reduced injury scores compared to IRI alone: 3.5 (1.25–5, *p* = 0.002) and 4.25 (4–5, *p* = 0.031) vs. 5.5 (4–6). RIC 48 h prior to IRI did not reduce injury but injury was lowest in early+preRIC group [2.375 (1.625–3) 0.4375, *p* = 0.0001]. IFN-γ, IL-4 and IL-5 were increased following RIC.

**Conclusion:**

RIC reduces the extent and severity of intestinal injury. Two doses of RIC delivered 48 h apart has the greatest effect. Cytokines IFN-γ, IL-4 and IL-5 may be important in the transmission of RIC.

**Supplementary Information:**

The online version contains supplementary material available at https://doi.org/10.1007/s00383-026-06717-8.

## Introduction

Necrotising enterocolitis (NEC) is a devastating intestinal disease with a high mortality, significant morbidity for the survivors [1] and a distinct lack of specific treatments. Whilst the precise pathophysiology of NEC is complex, ischaemia-reperfusion injury (IRI) is part of this process [2]. Remote ischaemic conditioning (RIC) is an endogenous phenomenon whereby the application of short cycles of non-injurious ischaemia and reperfusion to one organ system provide a systemic protective effect against ischaemia-reperfusion injury in a different organ [3]. We have previously reported the protective effect of remote ischaemic pre-conditioning (pre-RIC) in an animal model of NEC based on ischaemia-reperfusion injury to the intestine of rat pups [4]. Whilst the model does have certain limitations when used to replicate human NEC, it is well established and has been previously used for the study of putative therapeutic options for NEC and led to translational studies [5]. Furthermore, transcriptomic analysis of the model shows that the ischaemic pathways in the intestine have significant overlap with the inflammatory pathways known to be important in NEC [6].

One of the main limitations to the translation of RIC to clinical practice is the unpredictability of ischaemic diseases. Although there is good experimental data to suggest RIC may be protective against myocardial ischaemia, in clinical practice the timing of myocardial infarction is difficult to predict limiting the potential therapeutic utility of pre-RIC in this setting [7]. In the paradigm of NEC, RIC could be much more applicable for several reasons. Firstly, whilst NEC is an acute illness, its development is not entirely unpredictable; it is possible to identify high risk groups of preterm infants and these may be considered for future translation of pre-RIC in a preventative way. Secondly, the intestinal injury caused by NEC is on-going process as demonstrated by the reparative changes seen in surgical resection specimens [8]. This raises the possibility that RIC applied after disease onset may have therapeutic benefit. Consequently there is an interest in understanding the temporal relationship between the application of RIC and its protective effect in this model of NEC. Specifically, if postRIC (RIC delivered after the onset of injury) reduces injury this would be meaningful in the context of babies with a diagnosis of NEC (Bell stage 1 or above). Conversely if the protective effect of preRIC can be shown to last for a prolonged period of time this would make the preventative use of RIC in at-risk neonates practicable.

In other contexts, it has been shown that RIC can potentially have a prolonged protective effect [9]. Similarly, the protective effect of RIC delivered after the induction of ischaemia (post-RIC) is well established [10]. Given these observations in other models, our purpose here is to establish the protective effect of RIC at different time-points as this will inform clinical protocols for any putative human translation.

## Methods

### Animal model of intestinal IRI [4, 11, 12]

Animal experiments were carried out with ethical approval from the local Animal Welfare and Ethical Review Body and according to the Animals in Scientific Procedures Act (APSA) 1986 and revisions (Project licence: PA813F125). This study is reported in accordance with the ARRIVE guidelines [13].

The model was implemented as previously reported [4] but briefly rat pups (age 10–13 days) were subjected to intestinal ischaemia-reperfusion injury induced with occlusion of the superior mesenteric artery (SMA) using a microvascular clip (S&T, AG, Switzerland) via laparotomy under inhalational general anaesthesia. Arterial occlusion was confirmed by the loss of visible pulsation in the arterial arcades and colour-change in the bowel, the abdomen was closed temporarily. Forty minutes later the abdomen was re-opened, the clip was removed, reperfusion was confirmed by the return of visible pulsation within the arterial arcades and hyperaemic colour change in the bowel, and the abdomen was again temporarily closed. Ninety minutes later animals were euthanized by exsanguination (cardiac puncture to obtain serum) whilst still under terminal general anaesthesia and intestinal samples were harvested. Control animals underwent laparotomy and dissection of the SMA without application of the microvascular clip. All animals underwent vital sign monitoring (heart rate and oxygen saturation) using a pulse oximeter on the forelimb. Normothermia was maintained in all animals using a heating blanket.

### RIC application and experimental groups

**Fig. 1:**
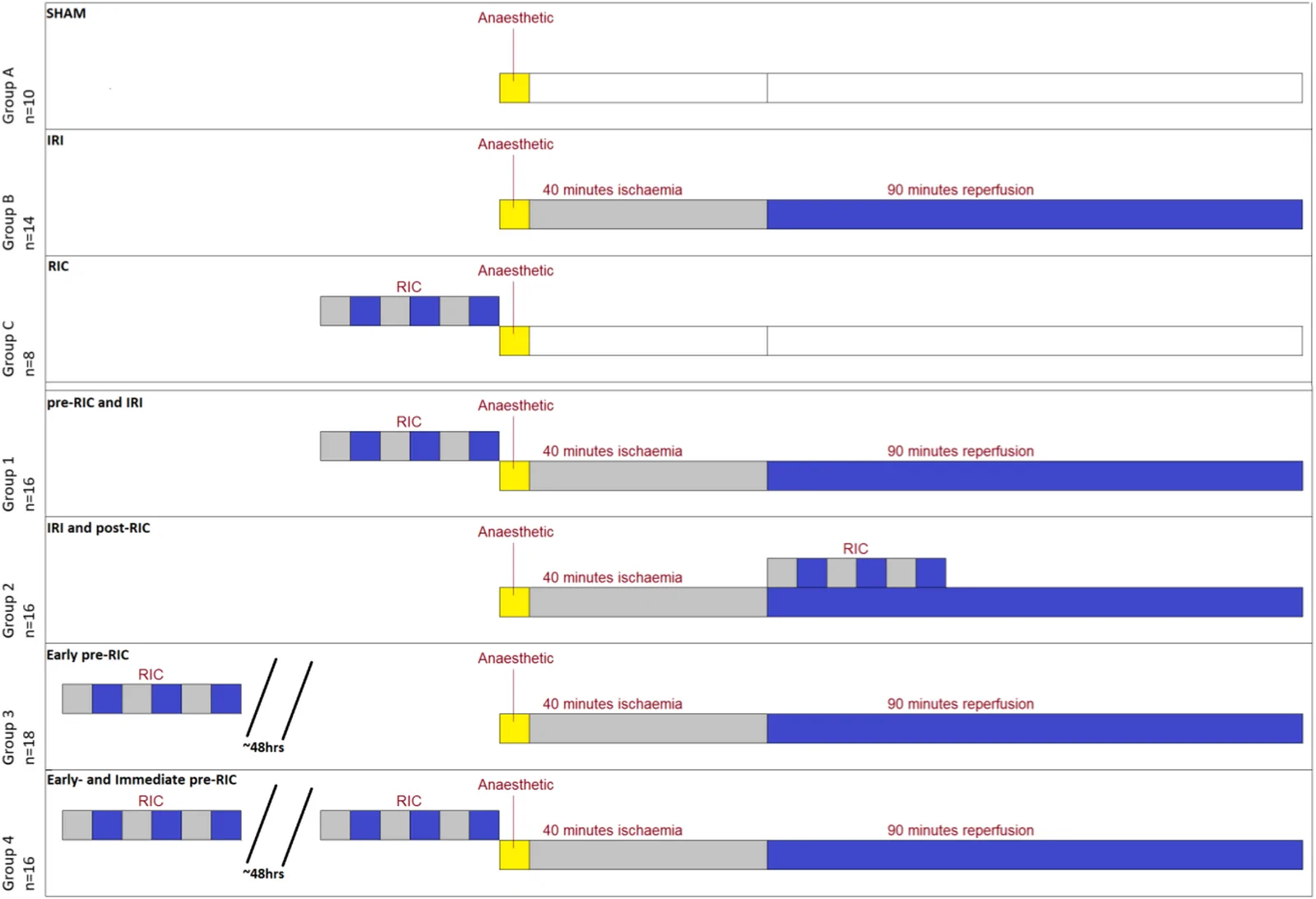
Time points for RIC and IRI for each experimental group. Rat pups were randomly allocated to each group. Group 1—preRIC (immediately prior to anaesthetic) as well as IRI; Group 2 -IRI with RIC at the time of reperfusion (postRIC); Group 3—RIC 48 h prior to injury (early preRIC); Group 4—two episodes of RIC: 48 h, and immediately prior to injury. Negative controls had sham surgery (group A); Positive controls had IRI alone (Group B); Group C had RIC and sham surgery

Rat pups from related litters were randomly assigned to each of the experimental groups shown in Fig. 1. Group 1 (*n* = 16), RIC applied immediately prior to injury (preRIC, previously reported); [4] Group 2 (*n* = 16), RIC applied at the beginning of the reperfusion phase (postRIC); Group 3 (*n* = 18), RIC applied 48 h prior to injury (early preRIC) and Group 4 (*n* = 16), RIC applied both 48 h and immediately prior to injury (early+preRIC). Negative controls had sham surgery (group A, *n* = 10). Positive controls underwent IRI only (Group B, *n* = 14). In order to gain further understanding into the potential mechanisms underlying the benefit conferred by RIC, a further group of rat pups underwent preRIC without IRI (Group C, *n* = 8).

Groupings were maintained for animals undergoing early preRIC by marking with a skin marker before returning them to their mothers for the time between early pre-RIC administration and delivery of the ischaemia reperfusion injury.

RIC was induced by means of a ligature applied to the hind limb. Occlusion of arterial inflow was demonstrated by a colour change in the limb. Each animal undergoing RIC received 3 cycles of 5 min of ischaemia with 5 min of reperfusion between ischaemia episodes. For all animals receiving RIC prior to IRI (i.e. preRIC and early preRIC groups), RIC was applied without general anaesthesia to avoid the previously reported potential confounding effect of general anaesthesia on the effect of RIC [14, 15].

### Macroscopic assessment of bowel injury

In all animals, immediately after euthanasia, the entire small bowel was removed and laid out straight. The bowel was assessed visually and sections were categorised as ‘normal,’ with no bowel injury, ‘severe injury’ if the bowel was frankly necrotic and ‘mild injury’ for any bowel that was affected but not frankly necrotic. The sections of bowel in each of these three categories were measured in centimetres and the total small bowel length was measured. Results are expressed as length of bowel in each injury severity category as a percentage of total small bowel length. This experimental model produces a variable pattern of intestinal injury with segments of frank necrosis, oedema and normal bowel seen side-by-side. Although macroscopic assessment was not blinded, macroscopic appearance correlates well with microscopic changes and hence measuring the proportion of bowel affected provides a meaningful measure of the extent of bowel injury [4, 16].

### Microscopic assessment of bowel injury

The Primary Outcome measure was the validated Chiu-Park Score [17, 18]. A 2 cm section of the most severely injured bowel was removed, flushed with phosphate buffered saline (with calcium chloride and magnesium chloride, (Gibco/Therma Fisher Scientific, USA)), cut into three pieces and then immediately fixed in 10% neutral buffered formalin. These three pieces were embedded in paraffin, 5 μm sections were cut and stained with haematoxylin and eosin. Each slide contained a section from each of the three pieces and thus was representative of the whole 2 cm sample. The samples were labelled with a randomly generated 3-digit code unique to the animal to allow blinded assessment by three assessors. As recommended by Quaedackers et al. [18] and Oltean et al., [19] the severity of bowel injury was assessed using the Chiu-Park scoring system [17] which gives a score of 0–8 based on specific features that indicate gradations of ischaemic bowel injury from no injury (score 0) to complete loss of villi and transmural infarction (score 8). The median Chiu Park score of the three assessors was used for to create a single score for each individual animal which was then used for analysis. This was done to avoid a Type I Error caused by duplication of data points. Spearman R coefficients were calculated in pairs between the three scorers. The lowest coefficient calculated was 0.72.

### Serum cytokine analysis

As described, the animals were euthanised by means of a cardiac puncture which allowed blood to be harvested. Whole blood was collected in tubes containing Ethylenediaminetetraacetic (EDTA), centrifuged at 3000 rpm for 5 min and plasma extracted, which was stored at − 80 °C prior to batch analysis. Plasma cytokine concentration was assayed using the MSD^®^ MULTI-SPOT Assay System (Proinflammatory Panel 2 (rat) kit (Meso Scale Discovery^®^, USA)). This assay measures IFN-γ, IL-1β, IL-4, IL-5, IL-6, KC/GRO (equivalent to human IL-8), IL-10, IL-13 and TNF-α in a single sample. Samples were thawed on the day of analysis and processed according to the manufacturer’s instructions; a dilution of 1 in 4 was used. This assay was chosen to allow simultaneous analysis of multiple pro-inflammatory cytokines in small sample volumes. Groups A, B, C, 1 and 2 are reported.

### Statistical analyses

Statistical analyses were carried out using Graphpad Prism version 9.5.1. Non-parametric, Mann-Whitney and Kruskal-Wallis tests (with post-hoc testing) were used as appropriate. Spearman r coefficients were calculated to evaluate correlations.The pre-RIC, IRI and SHAM groups have been previously reported. The various groups were run contemporaneously once we had established the model.

## Results

### Macroscopic assessment of intestinal injury

Both the total proportion of intestine showing injury and the proportion showing severe necrosis were markedly reduced in each of the groups that underwent RIC, regardless of the timing of RIC delivery.

In animals exposed to IRI alone the median proportion of the intestine showing injury was 100% (IQR 85–100%). In each of the groups exposed to RIC, the extent of injury was significantly reduced: in the preRIC group to 58% (15–84%, *p* = 0.008) and in the postRIC group 68% (56–80%, *p* = 0.0006). In the group that underwent RIC 48 h prior to injury the median proportion of the intestine showing injury was 68% (58–77%, *p* = 0.0004) and in the animals that underwent RIC both 48 h prior to injury and immediately prior to injury it was 76% (62–91%, *p* = 0.01).

**Fig. 2:**
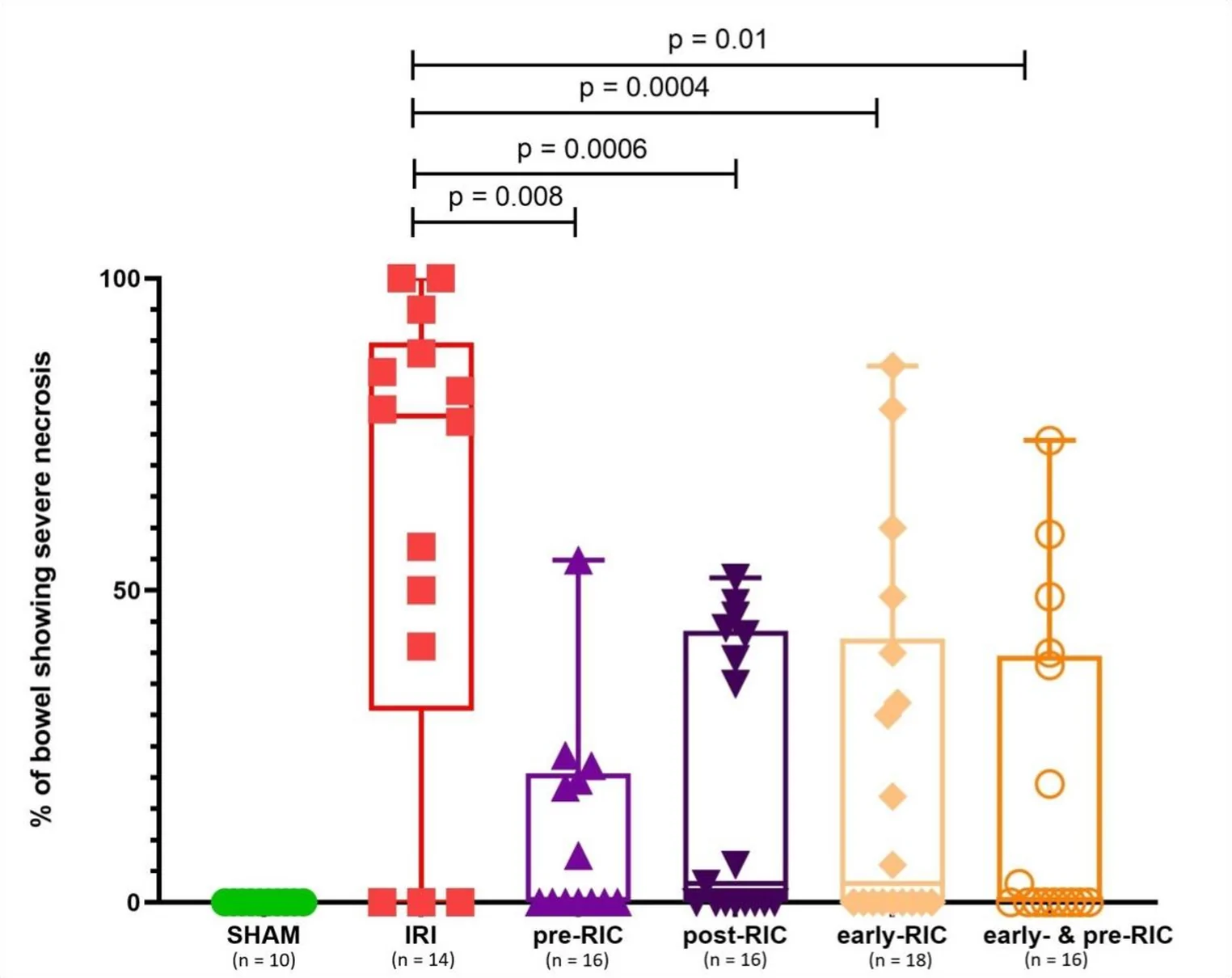
Percentage of intentine showing severe necrosis. Graph shows each data point, median, IQR and range for each group. Pups underwent sham surgery (negative controls); IRI alone (positive controls); IRI immediately prior to injury (preRIC); RIC during the reperfusion phase (postRIC); RIC 48 h prior to injury (earlyRIC) or both early- (48 h) and preRIC. p-values calculated with Mann-Whitney test compared to the positive controls

Assessment of the extent of severe necrosis showed similar results (Fig. 2). In animals exposed to IRI alone, severe injury involved a median 78% of the small intestine. This reduced to 4% in the preRIC group, 4% in the postRIC group, 3% in the early preRIC group and 0% in animals that had early+preRIC.

### Microscopic assessment of intestinal injury

**Fig. 3:**
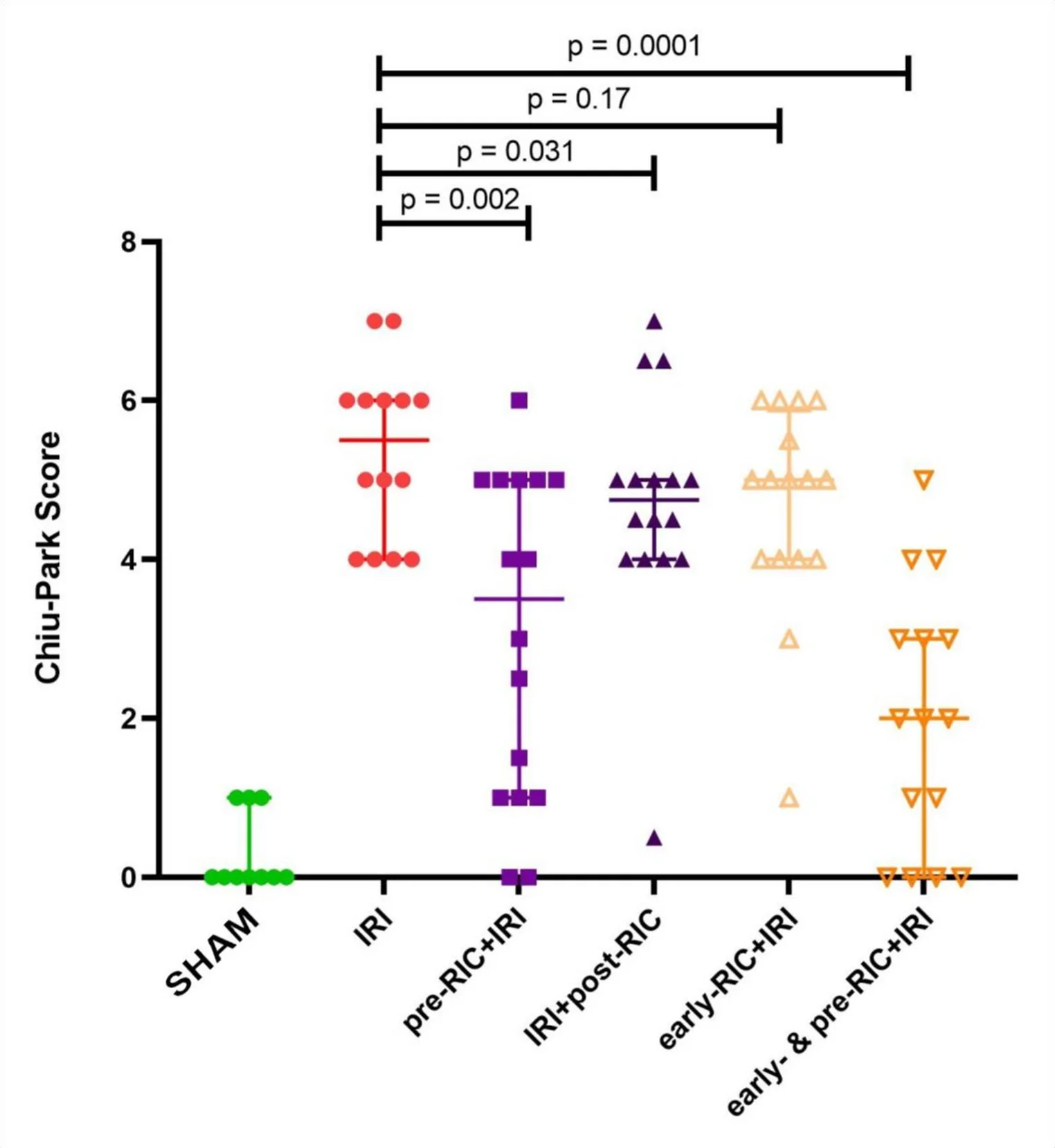
Chiu-Park scores of all groups. Graph shows each data point, median, IQR and range for each group. p-values calculated with Mann-Whitney Test. Comparison in each case is with the positive control: animals exposed to IRI alone. PreRIC + IRI, IRI+postRIC and early+preRIC + IRI all showed a statistically significant reduction in injury compared to IRI alone

Animals exposed to IRI alone had mean intestinal injury score of 5.5 (IQR 4–6), Animals that underwent SHAM surgery had a median score of 0 (Fig. 3). Compared to IRI alone, the application of RIC immediately prior to IRI and immediately following IRI (post-RIC) reduced this score to median 3.5 and 4.25 respectively. Application of RIC 48 h prior and immediately prior to injury had the lowest score of 2.375 (1.625–3.4375, *p* = 0.0001 vs. IRI alone). However, application of RIC 48 h prior to injury did not result in a statistically significant difference in injury score when compared to IRI alone.

### Serum pro-inflammatory cytokine levels

Serum cytokine levels at the end of the experiment were measured in Groups A, B, C, 1, and 2. Full results of cytokine analysis are detailed in supplementary material.

#### Cytokine changes associated with IRI

**Fig. 4:**
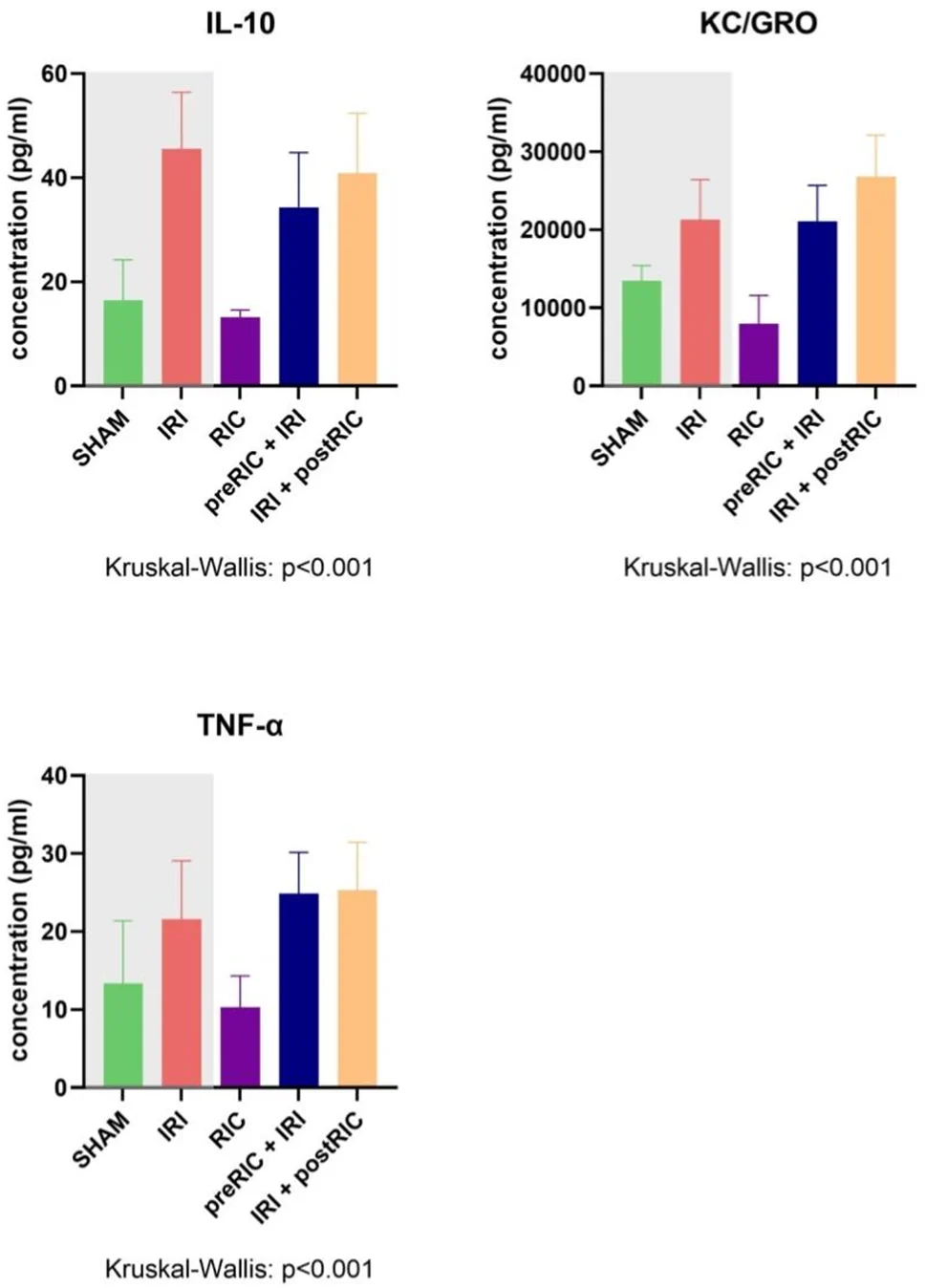
Serum concentration of Interleukin-10, KCL/GRO (Interleukin-8 equivalent) and Tumour Necrosis Factor alpha. Graph shows Median and IQR for each group. Levels are low in those that did not have IRI and raised in each group that had IRI. Grey boxes indicate controls (both positive and negative)

Interleukin-10, KC/GRO (Interleukin-8 equivalent) and Tumour Necrosis Factor Alpha all showed a significant difference between animals exposed to IRI vs. controls. IL-10, KC-GRO (IL-8 equivalent) and TNF-α were all raised in the groups that were exposed to IRI (Fig. 4). IL-10 levels were lower in those that also underwent RIC. All three showed moderate correlation with the extent and severity of intestinal injury (Table 1).

**Table 1:** Correlations between serum cytokine levels and intestinal injury

Correlation between intestinal injury and cytokines		Severe injury	Total injury	Microscopic injury score
Spearman r (95% C.I.) p-value	IL-10	0.47(0.23–0.65) **0.0002**	0.48(0.24–0.66) **0.00004**	0.46(0.22–0.64) **0.0003**
	KC-GRO	0.42(0.18–0.62) **0.001**	0.54(0.33–0.71) **0.0000009**	0.53(0.31–0.70) **0.00002**
	TNF-α	0.47(0.24–0.65) **0.0002**	0.49(0.26–0.67) **0.000009**	0.39(0.15–0.60) **0.002**

Spearman r coefficients with 95% confidence intervals and p-values reported

#### Cytokine changes associated with RIC

**Fig. 5:**
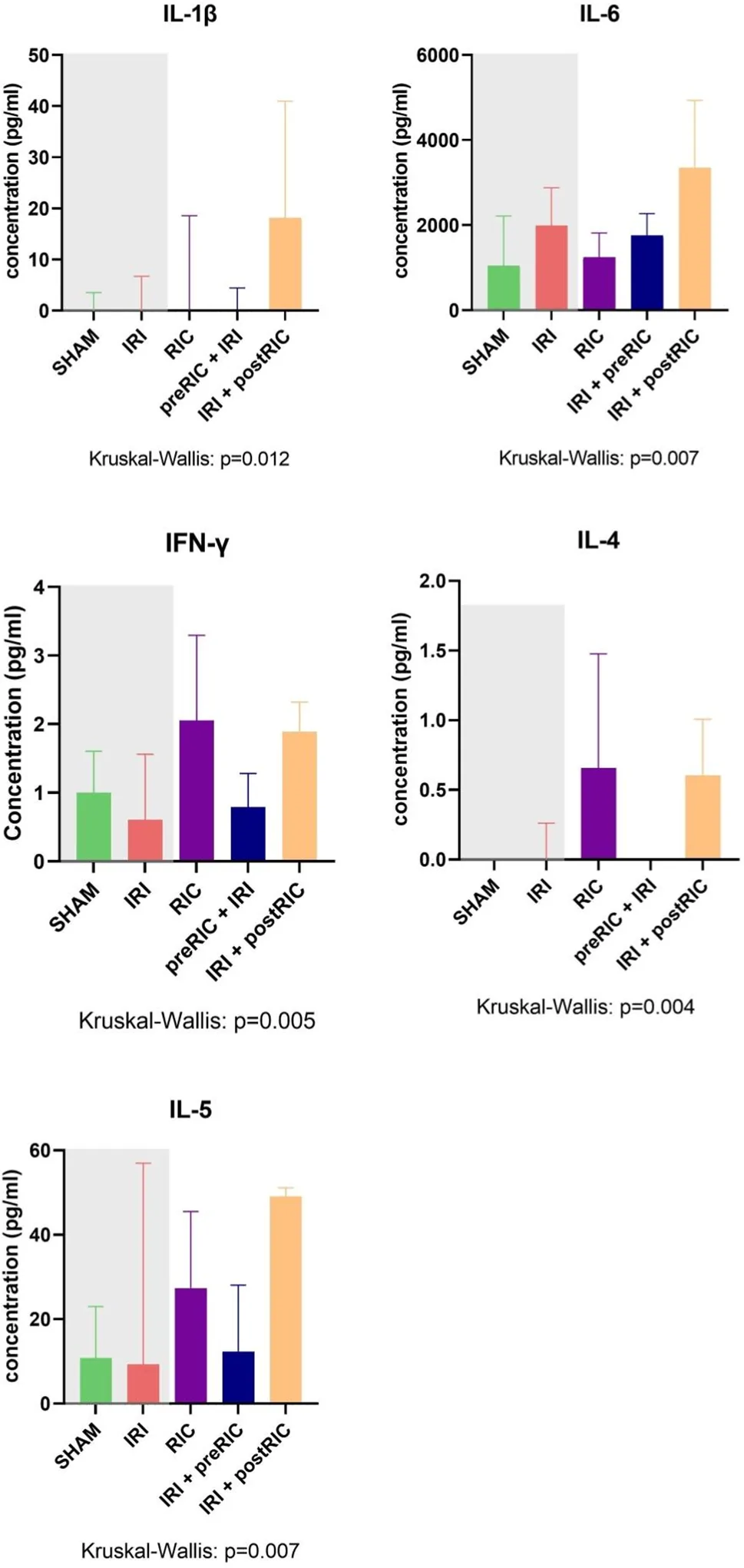
Serum concentration of Interleukin-1 Beta, Interleukin-6, Interferon-gamma, Interleukin-4 and Interleukin-5 Graph shows Median and IQR for each group. IL-1β and IL-6 were raised in the post-RIC group. IFN-γ, IL-4 and IL-5 were raised in both the RIC only group and the post-RIC group

In the post-RIC group, Interleukin-1-beta and Interleukin-6 were raised compared to the SHAM group (*p* = 0.03 and *p* = 0.006 respectively, Kruskal-Wallis, post-hoc comparison, Fig. 5). This group are the only ones whereby the serum levels were measured only 60 min after RIC (Fig. 1).

Interferon-gamma, Interleukins − 4 and − 5 showed higher levels in both the RIC-only group and the post-RIC group. This effect was particularly marked for IL-4 (*p* = 0.05 and *p* = 0.05 respectively, Kruskal-Wallis, post-hoc comparison, Fig. 5).

## Discussion

The need for novel therapies for NEC is evident from the fact that infants who contract the disease continue to have poor outcomes, both in terms of mortality and morbidity [1]. Previously we reported how the application of RIC just prior to injury resulted in a profound reduction of intestinal injury in this experimental animal model [4]. Work in other models has also shown that RIC has huge potential for reducing intestinal injury in NEC [20, 21]. A protocol for an early human trial of RIC in infants with NEC has been published [22]. Our focus in this study was on the temporal relationship between RIC application and the injury in order to inform future clinical trial design and thereby inform human translation. In this context, a prolonged effect of RIC is particularly important as it opens up the possibility of a practicable means of using RIC to prevent NEC.

Our results demonstrate that RIC was effective at reducing extent of macroscopic intestinal injury at all timepoints studied. When considering intestinal injury assessed microscopically (our primary outcome) RIC significantly reduced severity of intestinal injury in all experimental groups with the exception of early-preRIC. This supports the hypothesis that there is a temporal relationship between the timing of RIC application and the onset of IRI that influences the benefit conferred by RIC. RIC applied immediately prior to IRI or concurrent with reperfusion phase of injury resulted in a reduced severity of injury both macro and microscopically. However, RIC applied 48 h prior to injury alone reduced extent of macroscopic injury only (*p* > 0.06 for microscopic results). Interestingly, when RIC was applied repeatedly − 48 h prior to injury and again immediately prior to injury - the reduction in severity was the greatest, perhaps suggesting a cumulative effect of repeated ‘doses’ of RIC.

A prolonged protective effect of RIC has been postulated previously [9, 23], although it has received relatively little study [26]. Rohailla et al. [24]. studied the cardioprotective effect of RIC and showed that providing the RIC stimulus 24 h prior to injury was effective [24]. To the best of our knowledge, a prolonged protective effect has not been explored in the newborn intestine before. The effect of multiple applications of RIC has also been examined before. Menting et al. [15] showed no added benefit from repeated episodes. However, several other studies, in both humans and animal models have shown an increased benefit from repeated applications, specifically in the context of cardioprotection [25, 26]. Overall our data suggest that a clinically meaningful reduction in injury can be achieved with the application of RIC up to at least 48 h prior to injury. Furthermore, they provide evidence to support a ‘dose effect’ of RIC when applied 48 h apart. Whether this 48 h interval is optimal or whether intervals of a different duration would confer a different degree of benefit requires further investigation and is beyond the scope of this report.

Considering the context of clinical translation of RIC into human infants with or at risk of developing NEC our findings would support a role for RIC to be explored. Whilst the onset of timing of NEC in preterm infants who develop the disease is unpredictable, our findings demonstrate a beneficial effect when RIC is applied early after the onset of ischaemia (postRIC, group 2). In the clinical setting this would support a role for RIC even if applied soon after the disease had been clinically identified. Indeed a protocol for an early human trial of RIC in infants with NEC that mimics this scenario has been published [22]. Furthermore, our finding that the protective benefit of RIC appears to last for some time (up to 48 h) opens the possibility of RIC being utilised as a preventative intervention. Given that RIC has recently been demonstrated to be well tolerated in preterm infants [27], repeated applications of RIC at 48 h intervals in infants at high risk of NEC may be worthy of exploration in a controlled setting.

Perhaps the population for whom these findings have the greatest direct application for is those infants with congenital heart disease (CHD) who are at risk of developing NEC following surgical repair of their CHD on cardiorespiratory bypass. These infants represent a group in whom the timing of intestinal ischaemia (i.e. onset of bypass) can be predicted and open the possibility for applications of RIC at a precise time in relation to this. Several studies have examined the use of RIC in CHD but none have examined NEC risk. These studies reported no adverse effects of RIC [28–32]. Across all these populations it is also important to recognise that whilst our focus has been on the impact of RIC on ischaemic-reperfusion based organ injury, there has, in recent years, been significant interest in RIC as an anti-inflammatory process and not simply an ‘anti-IRI’ mechanism [33]. Increasing evidence suggests significant cross-over in the biochemical pathways [5, 34, 35]. This combined with the evidence from other NEC models [6, 20] gives a strong rationale for translation of RIC in all these populations.

### Serum cytokine analysis

The use of multiple time-points for the application of RIC, provides some insight to systemic cytokine responses to both the stimulus of IRI and RIC. IL-10, KC/GRO and TNF-α are raised in animals that suffered intestinal injury (both those that did and did not have RIC). The levels of these cytokines correlates with the extent and severity of intestinal injury. It has previous been shown that these all rise in human NEC [36]. KC/GRO is the rodent equivalent of human IL-8 and is a neutrophil chemoattractant [37]. It is readily produced in the premature gut of humans and is raised both locally and systemically in NEC [36]. More recently, it has been shown in human infants that IL-8 correlates with the degree of intestinal involvement in NEC [38]. Tissue and systemic TNF-α increases in NEC as well as other IRI diseases [36]. Collectively these data are consistent with this experimental model mirroring the human disease. However, these increases were not ameliorated by the application of RIC.

Animals in these experiments were exposed to two different interventions—IRI and RIC—making interpretation of the cytokine results complex and the following results should be interpreted with caution. The study was not powered for these analyses and the results need formal confirmation with a different study design that allows serum collection at multiple time points. The postRIC group are the only ones that had RIC only 60 min prior to the measurement of the serum cytokine level (Fig. 1, the other groups underwent RIC at least 130 min prior to sampling). As such, the rise in IL-1β and IL-6 seen in the IRI+postRIC group may be an effect of RIC specifically that is not seen in the RIC only and the preRIC + IRI groups (Fig. 5) because it could be temporary and by the time the serum was collected the peak of these has passed. It is particularly notable with IL-1β as the levels are negligible in all the other groups. In experimental models of NEC, IL-1β it has been shown to be raised in gut tissue and to promote Toll-like receptor 4 (TLR4) [39]. IL-6 is secreted mainly by T cells and macrophages and rises are seen after ischaemia-reperfusion injury [40]. Increased levels of IL-6 are seen in NEC at the tissue level [36] Higher levels of IL-6 have been seen in response to ischaemia and these levels are increased further by the application of remote pre-conditioning [41]. These data suggest that RIC alone can induce a rise in IL-6.

Interferon-gamma, Interleukin-4 and Interleukin-5 levels are also shown in Fig. 5. In each case there is a higher level in the postRIC group (similar to IL-1β and Il-6) but also a peak in the RIC only group. This strongly supports the notion that each of these cytokine rises in response to RIC. IFN-γ is a potent activator of macrophages. Previously serum IFN- γ has been shown to decrease in response to RIC [42]. IL-4 is produced by TH_2_ cells. It activates B-cells and inhibits macrophages [43]. It also supresses IFN-γ production. There is some evidence that IL-4 is protective against NEC [44]. Previous studies suggest IL-4 expression may be increased by RIC [45]. No previous studies have shown a role for IL-5 in the mechanisms of RIC.

Formally, it would require a different study design to evaluate these findings further. The rat pups small size means that it is only possible to collect serum samples of sufficient volume by cardiac puncture and thus multiple sampling from the same animal at different time-points is not practicable. Further elucidation of the cytokine pathways of RIC are clearly warranted and likely to need a very different (larger) animal model with opportunities for blood sampling at multiple time points.

There remain important limitations to this work. This model of intestinal injury does not fully represent human NEC. Although it has been demonstrated that the histological injury is very close to the human disease [46] as are the systemic effects [6, 47]. Gavage based models have benefits in this area but do not allow the temporal analysis done here. The pre-RIC was done prior to anaesthesia as this is a well-described confounder. This was not possible for the post-RIC group (nor could it be in the context of this model without compromising the ethical framework for animal experimentation. The evidence suggests that anaesthesia reduces the effect of RIC rather than increasing it. Every effort was made to minimise potential confounders and the animals were regularly handled but pups who did not undergo pre-RIC were not separated from the dams.

## Conclusion

In this intestinal IRI based model of NEC in newborn rats, we have identified a beneficial effect of RIC when administered at a range of timepoints including 48 h prior to, and immediately after the onset of ischaemia. These data provide a basis for clinical translation of RIC as either a treatment or preventative intervention in infants with - or at risk of developing - NEC. Analysis of serum cytokines suggests a role for a number of cytokines in the transmission of RIC.

## Electronic Supplementary Material

Below is the link to the electronic supplementary material.

Supplementary Table: Serum cytokine levels. Median values and inter-quartile range reported.

## Data Availability

All of the data in this report are part of a PhD thesis. The data is publicly available at: https://eprints.soton.ac.uk/475296/.
