# Supplementary material for: Post remote ischaemic conditioning and early pre remote ischaemic conditioning reduces the extent and severity of bowel injury in an animal model of necrotising enterocolitis
Source: Pediatr Surg Int. 2026 Nov 13;43(1):17. doi: 10.1007/s00383-026-06717-8 (PMC13768212; doi:10.1007/s00383-026-06717-8)
Supplement: Supplementary file 1 [file 383_2026_6717_MOESM1_ESM.docx]

|  | **SHAM** | **IRI** | **RIC only** | **preRIC** | **postRIC** |
| --- | --- | --- | --- | --- | --- |
| **IFN-γ**  pg/ml | 1.00  (0 - 1.6) | 0.61  (0 - 1.6) | 2.3  (2.1 - 4.1) | 0.79  (0 - 1.3) | 1.89  (1.3 - 2.3) |
| **IL-1β**  pg/ml | 0  (0 - 3.5) | 0  (0 - 6.7) | 18.44  (10.3 - 33.3) | 0  (0 - 4.4) | 27.8  (3.3 - 41) |
| **IL-10**  pg/ml | 16.47  (11 - 24.2) | 45.5  (28.3 - 56.4) | 14.29  (11.5 - 20.9) | 34.31  (27.6 - 44.8) | 26.29  (21 - 48.1) |
| **IL-13**  pg/ml | 3.95  (2.4 - 5.2) | 2.77  (1.5 - 4.5) | 4.11  (2.6 - 4.6) | 2.64  (1.4 - 3.9) | 5.02  (4.4 - 6) |
| **IL-4**  pg/ml | 0  (0 - 0) | 0  (0 - 0.2) | 0.98  (0.7 - 1.9) | 0  (0 - 0) | 0.23  (0 - 1) |
| **IL-5**  pg/ml | 10.81  (0 - 23) | 9.31  (0 - 57) | 38.09  (18.2 - 46.5) | 12.37  (0 - 28.1) | 44.7  (31.9 - 50.3) |
| **IL-6**  ng/ml | 1.05  (0.4 - 2.2) | 1.99  (0.8 - 2.9) | 0  (0 - 0) | 1.76  (1.3 - 2.3) | 3.35  (2.2 - 4.9) |
| **KC/GRO**  ng/ml | 13.47  (12 - 15.4) | 21.32  (18.4 - 26.4) | 0.01  (0 - 0) | 21.08  (16.7 - 25.7) | 26.8  (25.1 - 32.1) |
| **TNF-α**  pg/ml | 13.36  (10.2 - 21.4) | 21.63  (16.4 - 29.1) | 12.49  (8.4 - 18) | 24.88  (15.6 - 30.2) | 25.33  (20.2 - 31.5) |
| Supplementary Table: Serum cytokine levels. Median values and inter-quartile range reported. | | | | | |
